# The Mediating Effect of Social Networks on the Impact of Health Perceptions on the Quality of Life in Older Adults

**DOI:** 10.3390/healthcare13020122

**Published:** 2025-01-09

**Authors:** Selma Durmuş Sarıkahya, Amine Terzi, Yalçın Kanbay, Sevil Çınar Özbay

**Affiliations:** 1 Department of Public Health Nursing, Faculty of Health Sciences, Artvin Coruh University, Artvin 08000, Türkiye; 2 Department of Internal Medicine Nursing, Faculty of Health Sciences, Artvin Coruh University, Artvin 08000, Türkiye; deniz.amine@artvin.edu.tr; 3 Department of Psychiatric Nursing, Faculty of Health Sciences, Artvin Coruh University, Artvin 08000, Türkiye; yalcinkanbay@gmail.com; 4 Faculty of Health Sciences, Artvin Coruh University, Artvin 08000, Türkiye; sevilcinar@artvin.edu.tr

**Keywords:** older adults, social network, health perception, health-related quality of life

## Abstract

**Background/Objective:** Social networks help improve psychosocial and quality-of-life outcomes among older adults. This study aimed to examine the mediating role of social networks in the effect of health perception on the quality of life of the elderly. **Methods:** The sample of the study consisted of 327 people over the age of 65 who applied to a family health center. The study data were collected using the “Personal Information Form”, “Perception of Health Scale”, “World Health Organization Quality of Life Instrument-Older Adults Module”, and “Lubben Social Network Scale”. Multivariate regression analyses and mediation effect examinations were conducted to explore the relationships between social networks, health perception, and quality-of-life outcomes. **Results:** According to the findings, social networks mediate the relationship between health perception and quality of life. Individuals with a high level of health perception and a high level of social networks have higher quality-of-life levels than others. **Conclusions:** The results of the study confirm the significant correlation between health perception and the quality of life, as well as any potential links between these factors and social networks that affect older people’s quality of life.

## 1. Introduction

The number and proportion of elderly individuals aged 65 and over is rapidly increasing worldwide. The decline in the health of the elderly and the social and psychological effects of aging are becoming social problems along with the phenomenon of an aging society across the globe [1]. In Turkey, the elderly population rate is 10.2%, and according to population projections, this rate is expected to reach 12.9% in 2030, 16.3% in 2040, 22.6% in 2060, and 25.6% in 2080 [2].

One of the main factors affecting the quality of life of the elderly is their health status. With aging, the frequency of physical and mental health problems increases, which negatively affects the health perception of the elderly. Social health determinants can influence such health declines [1]. Social relationships and social participation are important social determinants that significantly impact health [3]. These social determinants fall under the category of a “social network” and are among the key factors affecting the quality of life of elderly individuals [4].

Social networks can consist of various social relationships with close friends, family members, neighbors, and others in the surrounding environment [1]. Social networks are considered precursors to social support and provide a model or pathway for it to emerge [5]. Insufficient or nonexistent social networks can lead to social isolation, which increases health risks [6]. Studies have linked the absence of social networks to negative health outcomes, including loneliness, depression, low self-esteem, disability risk, and premature death [6,7].

Although a reduction in social networks is a natural result of aging, the size of elderly individuals’ social networks and the frequency of communication with network members can directly impact their health and overall well-being. For elderly individuals, social networks allow them to strengthen bonds with loved ones, meet new people, expand their social circles, and access social support mechanisms more easily. In this context, elderly individuals feel more valued, safer, and healthier, which positively affects their health perception. Additionally, strengthening social bonds reduces feelings of loneliness, creating a positive impact on life attachment and psychological well-being [8,9,10].

A South Korean study revealed a significant relationship between social networks and health-related quality of life, potentially influencing the health-promoting behaviors of elderly Korean adults [1]. The presence of social relationships contributes to lifelong healthy living, and the quality of these relationships is a determining factor in health. High-quality supportive relationships have positive effects on health, well-being, adherence to medical treatments, and avoidance of negative behaviors, while low-quality or tense relationships can have adverse effects on health [11,12].

Elderly individuals may experience a reduction in important social relationships as they face losses, job changes, the death of a close partner or peers, poor health, and societal changes [13,14]. Loss of size in social networks or a decrease in communication frequency may reduce elderly individuals’ general physical and cognitive functionality, making it harder for them to cope with losses, hindering regular exercise, and negatively impacting their health [15]. On the other hand, elderly individuals with strong and participatory social networks can promote healthy aging by increasing their health and well-being through health-promoting behaviors like physical activity, excellent nutrition, and stress management [1].

Participation in a newly opened community center was associated with significant improvements in self-reported health over time in a quasi-experimental intervention study on elderly individuals [16]. Researchers in Italy discovered a decline in health-related quality of life, as measured by mental and physical health indicators, when friends interacted less frequently [17].

The presence of social relationships in elderly individuals improves their mental health by providing attachment, a sense of belonging, and a sense of purpose in their lives. Strong social networks and social interactions with family and friends have been found to be related to quality of life [18,19], while a decrease in the frequency of social interactions leads to a lower quality of life [20].

Research has reported that health perception significantly influences the quality of life of elderly individuals [21,22]. Individuals’ evaluations of their general health status shape health perception, reflecting the biological, mental, and social dimensions of their health [23]. With aging, the prevalence of chronic diseases such as diabetes, hypertension, arthritis, and heart disease increases, which may negatively affect health perception due to physical function decline or pain. Likewise, health problems like mobility restrictions, vision, and hearing loss can also negatively impact health perception. Additionally, cognitive declines such as depression, anxiety, and dementia can adversely affect health perception [20,21,22]. Studies have found that elderly individuals who perceive their health positively tend to have a higher quality of life [22]. Choi and Lee (2019) stated that elderly individuals who perceive their health as excellent have a higher quality of life [24]. Jung et al. (2012) emphasized that subjective health status is an important variable affecting quality of life [25]. The healthier elderly individuals feel, the more positive their thoughts about life are, the more satisfied they are with it, and their quality of life is higher [26].

Even if elderly individuals have chronic illnesses, those who perceive their health positively are more resilient and possess better coping mechanisms, while those who perceive their health negatively tend to have a lower quality of life. This perception can lead to feelings of helplessness, withdrawal from social activities, and a loss of motivation for health-promoting behaviors [22,23].

Many studies confirm the contributions of health perception and social networks to life expectancy and life satisfaction in elderly individuals [1,26,27,28]. However, studies on the determinants of quality of life in the elderly indicate that no single factor is effective, and there are still some debates about the exact nature of these relationships [7,29].

Given the increasing life expectancy in developed countries, understanding the determinants of health and quality of life in the aging process is of enormous importance [30,31]. There is a need for studies on how different aspects of healthy aging translate into perceived health and quality of life. For societies, the challenge is not only to ensure healthy aging but also to enable individuals to perceive good quality of life as they age healthily. While the literature tries to reveal the relationship between health perception and quality of life [21,22,25], there is no study on the mediating role of social networks. Traditionally, elderly individuals’ social network structures influence their self-perception, which forms the basis of the indicators used to measure their quality of life. There is still a lack of research on the factors influencing the social network structures and quality of life of elderly individuals [31].

Socio-economic shifts such as industrialization, urbanization, and individualization have been transforming Turkey’s traditional extended family structure, which traditionally maintained strong intergenerational ties and relied on families, children, or grandchildren to care for elderly individuals. The rise in the nuclear family model and the weakening of the extended family structure have resulted in negative effects such as loneliness, social isolation, and poor health perception among elderly individuals. This is particularly evident when young people relocate to different cities for education and work, leading to a shift in the responsibility for elderly care [32].

This study makes a significant contribution to the literature by examining the impact of elderly individuals’ social networks on their health perception and quality of life. It helps us better understand the social support needs of older adults. Furthermore, by emphasizing that strengthening social networks could be an important strategy for improving the health perception and overall quality of life of elderly individuals, the study aims to contribute to the development of social policies and intervention programs.

## 2. Materials and Methods

### 2.1. Purpose of the Study

This study aimed to examine the relationship between health perception and quality of life in older adult individuals and the mediating role of social networks in this relationship.

### 2.2. Study Design and Hypotheses

This study employs a relational research design which aims to examine the relationships and/or the level of covariance between two or more variables [33]. In line with the purpose of the study, models and hypotheses regarding the mediation role are given below.

**H1.**  *“Health Perception” has an impact on “Social Network”.*

**H2.**  *“Social Network” has an impact on “Quality of Life”.*

**H3.**  *“Health Perception” has an impact on “Quality of Life”.*

**Figure 1:**
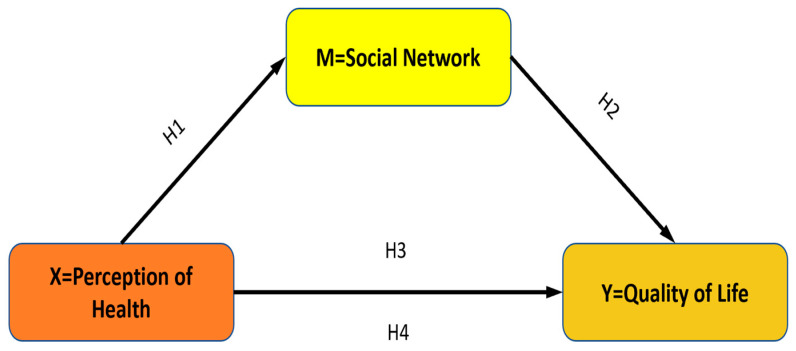
Theoretical structure of the mediation model.

**H4.**  *In the relationship between “Health Perception” and “Quality of Life”, the “Social Network” is a mediating variable (Figure 1).*

### 2.3. Sample of the Study

This study was conducted at a family health center located in the central district of a province in the Central Anatolia Region of Turkey between October and November 2023. There are a total of 201 family health centers in this district. One family health center was randomly selected from these 201 centers, and the study was conducted at this center. The total number of individuals aged 65 and older at the selected family health center is 2150. The sample size, calculated with a 95% confidence level and a 5% margin of error, is 327 individuals. The study includes 327 elderly individuals who visited the selected family health center during the specified period.

Inclusion criteria:

- Participants must be aged 65 and older.
- No communication barriers should be present.
- Willingness to participate in the study.

Exclusion criteria:

- The data collection forms contain incomplete or incorrect information.
- Wanting to leave the study at any stage of the study.

### 2.4. Data Collection Tools

The researchers collected the study data using the “Personal Information Form”, “Perception of Health Scale”, “Lubben Social Network Scale (LSNS-6)”, and “World Health Organization Quality of Life Instrument-Older Adults Module (WHOQOL-OLD)” [34,35,36]. The participants responded to the questions on a voluntary basis.

#### 2.4.1. Personal Information Form

The researchers prepared the Personal Information Form, which includes some sociodemographic characteristics of the participants.

#### 2.4.2. Perception of Health Scale (PHS)

The Perception of Health Scale (PHS) was developed by Diamond et al. (2007) [34] and adapted to Turkish culture by Kadıoğlu and Yıldız (2012) [37]. The PHS is a five-point Likert-type scale consisting of 15 items and four sub-factors. Items 1, 5, 9, 10, 11, and 14 are positive statements, while items 2, 3, 4, 6, 7, 8, 12, 13, and 15 are negative statements. We scored positive statements as “strongly agree = 5”, “agree = 4”, “undecided = 3”, “disagree = 2”, and “strongly disagree = 1”. Negative statements were reverse scored. The scale consists of four sub-dimensions. These dimensions are “Center of Control” (items 2, 3, 4, 12, and 13), “Certainty” (items 6, 7, 8, and 15), “Importance of Health” (items 1, 9, and 11), and “Self-Awareness” (items 5, 10, and 14). The scale yields a minimum score of 15 and a maximum score of 75. A high score indicates that the perception of health is positive.

#### 2.4.3. Lubben Social Network Scale (LSNS-6)

The Lubben Social Network Scale (LSNS-6) was developed by Lubben (1988) for older adult populations to measure social networks, including family and friends, and has been widely used in research and clinical settings [35]. Demir Erbil and Hazer (2020) conducted the Turkish adaptation study of the scale [38]. Each scale item was scored from 0 to 5 in a six-point Likert-type scale, as in the original; 0, 1, and 2 indicate less social engagement and 3, 4, and 5 indicate more social engagement. The total score is calculated by summing the scores obtained from all items. The scoring for LSNS-6 ranges from 0 to 30, with a higher score indicating a greater presence of social networks. The original scale yielded a reliability coefficient of 0.83 for the overall scale.

#### 2.4.4. World Health Organization Quality of Life Instrument—Older Adults Module (WHOQOL-OLD)

Within the scope of the development study conducted in 22 countries under the auspices of the World Health Organization Quality of Life Group (WHOQOL Group) [36], the WHOQOL-OLD was adapted to Turkish culture by Eser et al. (2010) [39].

It is a 5-point Likert-type scale consisting of 6 sub-dimensions and 24 items to assess the quality of life in elderly individuals. The sub-dimensions are sensory abilities (items 1, 2, 10, and 20), autonomy (items 3, 4, 5, and 11), past, present, and future activities (items 12, 13, 15, and 19), social participation (items 14, 16, 17, and 18), death and dying (items 6, 7, 8, and 9), and intimacy (items 21, 22, 23, and 24). The score that can be obtained from each sub-dimension varies between 4 and 20. A total score can also be calculated by summing each of the individual item values. Higher scores indicate a higher quality of life.

### 2.5. Data Analysis

We examined the study’s data using SPSS 26 package software. We examined the skewness and kurtosis values to determine the normality of the data, deeming the distribution normal for values below +1.5 [40]. We examined Cronbach’s alpha values to test the reliability levels of the measurement tools used in the study. In the evaluation of the data, numbers, means, and percentages were given for descriptive statistics. To examine the mediation effect, “Process Macro”, developed by Hayes (2017), was used, and Model 4 was selected [41]. The study used Pearson correlation analysis to interpret the relationships between the measurement tools.

## 3. Results

Table 1 presents the sociodemographic characteristics of the 327 elderly adults participating in the study. The mean age of the participants was 70.1 ± 7.2 years (min: 65; max: 90) and 50.5% were female. The majority were married (90.2%) and had completed primary education (65.1%). Among the participants, 83.2% lived with their spouses and 82% had at least one chronic illness. Additionally, 59.3% used assistive devices such as glasses, walking canes, or wheelchairs. In terms of employment status, 66.4% were retired, and regarding income level, 57.5% were in the middle-income group (Table 1).

Cronbach’s α reliability coefficients were examined for the reliability levels of the measurement tools. Cronbach’s α coefficients for PHS, WHOQOL-OLD, and LSNS-6 were calculated as 0.74, 0.80, and 0.73, respectively. These reliability coefficients show that all three scales used in this study have sufficient reliability (Table 2).

The mean PHS score was 46.32 ± 3.4, the mean WHOQOL-OLD score was 74.50 ± 7.6, and the mean LSNS-6 score was 16.43 ± 4.2 (Table 2). There was no statistically significant correlation between health perception and quality of life (r: 0.023; *p* > 0.05). A positive and significant correlation was found between health perception and social networks (r = 0.128; *p* < 0.05). When the correlation between quality of life and social networks was analyzed, similarly, the correlation was found to be positive and significant (r = 0.198; *p* < 0.001).

**Figure 2:**
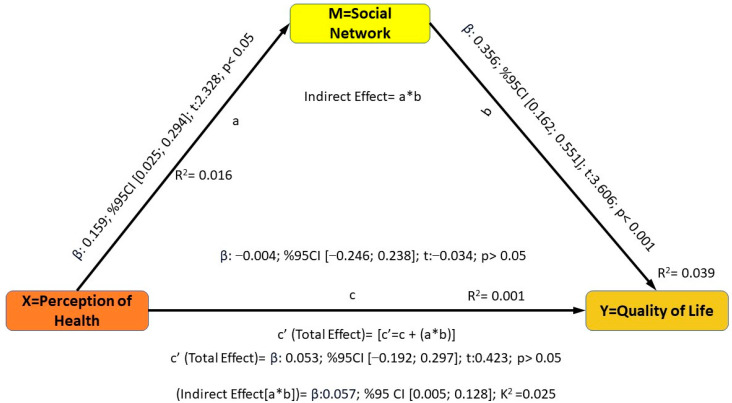
The mediating role of the social network in the effect of health perception on quality of life.

It was determined that the effect of “health perception (X)” on the mediating variable “social network (M)” was positive and significant (β: 0.159; 95% CI [0.025; 0.294]; t: 2.328; *p* < 0.05). According to these findings, as the level of “health perception” increases, the “social network” increases. A one-unit increase in the “health perception” score leads to a 0.159 unit increase in the “social network” variable (Figure 2). According to the data obtained, 2% (R^2^ = 0.016) of the variance in the “social network” level is due to the “health perception” variable. These findings led to the acceptance of the H1 hypothesis.

A study looked at how the variable “Social network (M)” affected the outcome variable “Quality of Life (Y)” and found that there was a positive and statistically significant path between them (β: 0.356; 95% CI [0.162; 0.551]; t: 3.606; *p* < 0.001). According to these findings, as the “Social Network” score increases, the “Quality of Life” score also increases. A one-unit increase in the “social network” variable leads to an increase of 0.356 units in the “quality of life” variable. According to the data obtained, 4% (R^2^ = 0.039) of the variance in the “Quality of life” variable is due to the “Social network”. These findings led to the acceptance of the H2 hypothesis.

When the total effect of the “Health perception (X)” variable on the “Quality of life” variable, which is the result variable, was examined, it was determined that this effect was statistically insignificant (β: 0.053; %95 CI [−0.192; 0.297]; t = 0.423; *p* > 0.05). In addition, the direct effect of “health perception” on the “quality of life” variable was not statistically significant (β: −0.004; 95% CI [−0.246; 0.238]; t: −0.034; *p* > 0.05). These findings indicate a lack of relationship between the health perception variable and the quality-of-life variable. These findings led to the rejection of the H3 hypothesis.

According to the findings, the indirect effect of “health perception” on “quality of life” is significant; thus, “social network” mediates the relationship between “health perception” and “quality of life” (indirect effect [a*b] = β: 0.057; 95% CI [0.005; 0.128]; K^2^ = 0.025). As a result of the bootstrap analysis, the bias-corrected and accelerated confidence interval (BCA CI) values do not include a value of 0 (zero). When interpreting effect sizes, if K^2^ = 0.01, it is interpreted as a low effect; if K^2^ = 0.09, it is interpreted as a medium effect; and if K^2^ = 0.25, it is interpreted as a high effect. The fully standardized effect size of the mediation effect is K^2^ = 0.025, and this value shows that the mediation role of the “social network” in the relationship between “health perception” and “quality of life” has a low level of effect.

Individuals with a high level of health perception and a high level of social networks have higher quality of life levels than others. These findings led to the acceptance of the H4 hypothesis.

## 4. Discussion

This study examined the mediating role of social networks in the effect of health perception on the quality of life in older adults. The findings indicate that the direct effect of health perception on quality of life was not significant, but social networks play an important mediating role in this relationship. An increase in health perception enhances social network levels, which in turn improves quality of life. This finding highlights the significant role of older adults’ social interactions and health perceptions in improving their quality of life.

This study revealed the positive relationship between social networks and quality of life. Abu Hammattah et al. (2021) reported that older adults who have large social networks and are in frequent contact with these networks have a higher quality of life [18]. In longitudinal cohort studies in Ireland, Ward et al. (2019) found that changes occurred in the quality of life of older adults over time, with the most significant changes observed in the dimensions of social networks and social activities, and that a decrease in social networks led to a decline in quality of life [42]. Previous studies show that social networks are one of the most significant determinants of quality of life. Hussain et al. (2023) reported that social networks help improve quality-of-life outcomes [8], while Kooshiar et al. (2012) reported that a decrease in the frequency of social interaction reduces quality of life [20]. In summary, these results confirm our hypothesis that social networks have a significant impact on the quality of life. Accordingly, the expanded network size and more frequent contact increase the chances of receiving effective social support, as it provides more social connection opportunities to the individual. In addition, having reliable social networks can protect individuals from insecurity and improve self-esteem, which can lead to improved quality of life [18].

The study found that older adults’ perceptions of their health were effective on social networks. Numerous studies demonstrate the connection between health perception and social networks [1,8]. Researchers report that physical health problems, chronic diseases, loss of muscle strength, physical limitations, and dependence on others complicate social interaction for older adults, causing them to narrow their social networks, distance themselves from social interactions, and ultimately experience feelings of loneliness and depression [43,44]. There is evidence that people with fewer social network ties are at higher risk of mortality and morbidity, suicidality, various diseases, disability, etc. [8], while those with adequate social networks and a supportive environment have higher levels of physical and mental health [44,45]. To prevent the development and exacerbation of health problems, especially in older people, it is necessary to know the importance of social networks and to increase the size and frequency of contact with social networks for healthy aging. In our study, social network measurement only measures participants’ contacts with family and friends. Therefore, we need future studies to quantify a broader definition of social networks.

This study concluded that health perception does not have an effect on quality of life. However, other studies in the literature have shown that health perception significantly affects the quality of life of older adults. For example, Mohamad Fuad et al. (2020) indicated that older adults’ self-perceptions regarding their general health affect their quality of life [21]. Choi and Lee (2019) found that older adults’ quality of life is higher when they believe they are healthy [24]. Additionally, many studies have proven that good health perception in older age is closely related to perceived quality of life, well-being, and life satisfaction [22,26]. These findings suggest that health perception is an important factor affecting older adults’ quality of life, but factors such as sample characteristics and local conditions may influence this relationship in different ways.

Studies in the literature have examined the relationship between health perception and quality of life through factors such as mental health, age, physical activity, and body image dissatisfaction [31,46,47]. However, there are no studies that address the mediating role of social networks in this relationship. The findings of our study show that health perception affects the quality of life in older adults through social networks. This suggests that social networks may have a significant impact on the quality of life of elderly individuals. Therefore, understanding the mechanisms by which different aspects of healthy aging influence perceived health and quality of life is of critical importance. Positive, high-quality, and supportive relationships may mitigate the negative effects of deteriorating health on quality of life, making this an important factor. In this context, understanding the impact of social networks on improving the health of older individuals is crucial for enhancing their quality of life and strengthening their health.

Consequently, maintaining social interactions and activities is essential at the heart of healthy aging, ensuring that older people continue to lead enjoyable lives even in poor physical or psychological health.

### 4.1. Limitations of the Study

Future research needs to address several limitations of this study. The participants of this study were selected from older adults in the central district of Kayseri province, which means that the results are specific to this geographic region and may not be applicable to other areas or populations. Therefore, we cannot generalize the findings to the broader elderly population in Turkey or other countries. Additionally, the study focused solely on the participants’ contacts with their families and friends when measuring the social network, which is only one component of a broader social network structure. Other aspects, such as community involvement, acquaintances, or interactions with other social groups, were not considered. This limited scope may have excluded factors that also influence the health perception and quality of life of elderly individuals.

### 4.2. Recommendations for Future Research

Future studies should explore the impact of interventions aimed at expanding and strengthening elderly individuals’ social networks, particularly focusing on individual programs and social support groups that enhance social empowerment. Research on how these interventions affect the quality of life of elderly individuals could provide valuable insights. Moreover, it would be beneficial to investigate the effects of educational programs and social activities that aim to increase the elderly’s self-esteem and enhance their health perception. These interventions may play a significant role in improving their overall well-being. Additionally, further research could examine other components of social networks, such as community involvement, to better understand their impact on health outcomes for older adults.

## 5. Conclusions

As a result, social networks are a significant variable in the effect of health perception on quality of life. The study confirmed a positive relationship between the perception of health and social networks. Based on both these study results and the literature data, it is necessary to maintain a social support network for healthy aging.

Considering the increasing elderly population in Turkey, it is important for health policymakers, healthcare professionals, and educators to develop programs aimed at preventing the disruption of social networks among elderly individuals. In this context, strategies should be developed to improve interpersonal relationships. To encourage and support the active participation of the elderly in social life, spaces should be created within the social structure where they can engage. For example, projects that provide income-generating opportunities, such as innovative initiatives, cooperative models, local markets, and fairs, will help elderly individuals participate more actively in social life and feel valued.

Additionally, giving elderly people active roles in cultural and tourist activities, such as storytelling, handicrafts, and agricultural tourism, will support their socialization and sharing of knowledge and experience with society. These types of activities can also help elderly individuals gain economic benefits. Social organizations, associations, clubs, hobby groups, and volunteer activities that enhance the freedom of life for the elderly and make them valuable actors in social life will increase their participation and support their contributions to society.

Finally, we believe that the findings of this study will serve as an important foundation for active aging policies in Turkey and provide essential data in the formulation of public policies. In this context, the development of policies that strengthen the social networks of the elderly and improve their overall quality of life is of critical importance for supporting healthy aging processes in society.

## Figures and Tables

**Table 1:** Sociodemographic characteristics of participants.

Characteristic		Total Participants (N = 327)
Age (years)	65–74	258 (78.9%)
	75–84	52 (15.9%)
	85+	17 (5.2%)
Gender	Male	162 (49.5%)
	Female	165 (50.5%)
Marital Status	Married	295 (90.2)
	Widowed/divorced/separated	30 (9.2%)
	Single (never married)	2 (0.6%)
Educational Level	No formal education	57 (17.4%)
	Primary school	213 (65.1%)
	Secondary school	51 (15.6%)
	Higher education	6 (1.8%)
Living Arrangement	Alone	26(8.0%)
	With spouse	272 (83.2%)
	With spouse and children	16 (4.9%)
	With children	8 (2.4%)
	Other (relatives, nursing home)	5 (1.5)
	Employed	18 (5.5%)
Employment Status	Retired	217 (66.4)
	Unemployed	92 (28.1%)
	Low	2 (0.6%)
Income Level	Medium	188 (57.5%)
	High	137 (41.9%)
Chronic Conditions (number of conditions)	0	34 (10.4%)
	1–2	268 (82.0%)
	3–4	25 (7.6%)
Use of Assistive Devices	No	133 (40.7%)
	Yes	194 (59.3%)

**Table 2:** Mean, distribution normality, correlation, and reliability findings of variables.

Variables	X	SD	Skewness	Kurtosis	1	2	3	Cronbach’s
**1. PHS**	46.32	3.4	0.242	0.334	1	0.023	0.128 *	0.74
**2. WHOQOL-OLD**	74.50	7.6	−0.324	1056		1	0.198 **	0.80
**3. LSNS-6**	16.43	4.2	−0.372	0.620			1	0.73

* *p* < 0.05; ** *p* < 0.001; PHS: Perception of Health Scale; WHOQOL-OLD: World Health Organization Quality of Life Instrument—Older Adults Module; LSNS-6: Lubben Social Network Scale.

## Data Availability

The data presented in this study are available upon request from the corresponding author.

## References

[1] Hong M., De Gagne J.C., Shin H. (2018). Social networks, health promoting-behavior, and health-related quality of life in older Korean adults. Nurs. Health Sci..

[2] (2023). TUİK Turkish Statistical Institute. https://data.tuik.gov.tr/Bulten/Index?p=Adrese-Dayali-Nufus-Kayit-Sistemi-Sonuclari-2023-49684.

[3] Heiman H., Artiga S. (2015). Issue Brief. Beyond Health Care: The Role of Social Determinants in Promoting Health and Health Equity.

[4] Kim B.J. (2014). Mediating effect of adult day health care (ADHC) and family network on quality of life among low-income older Korean immigrants. Res. Aging.

[5] Langford C.P.H., Bowsher J., Maloney J.P., Lillis P.P. (1997). Social support: A conceptual analysis. J. Adv. Nursing..

[6] Cornwell E.Y., Waite L.J. (2009). Social disconnectedness, perceived isolation, and health among older adults. J. Health Soc. Behav..

[7] Moreno-Tamayo K., Manrique-Espinoza B., Ramírez-García E., Sánchez-García S. (2020). Social isolation undermines quality of life in older adults. Int. Psychogeriatr..

[8] Hussain B., Mirza M., Baines R., Burns L., Stevens S., Asthana S., Chatterjee A. (2023). Loneliness and social networks of older adults in rural communities: A narrative synthesis systematic review. Front. Public Health.

[9] Montgomery S.C., Donnelly M., Bhatnagar P., Carlin A., Kee F., Hunter R.F. (2020). Peer social network processes and adolescent health behaviors: A systematic review. Prev. Med..

[10] Latkin C.A., Knowlton A.R. (2015). Social network assessments and interventions for health behavior change: A critical review. Behav. Med..

[11] Nicholson N.R. (2012). A review of social isolation: An important but underassessed condition in older adults. J. Prim. Prev..

[12] Stokes J.E., Moorman S.M. (2018). Influence of the social network on married and unmarried older adults’ mental health. Gerontologist.

[13] Seeman T.E. (2000). Health promoting effects of friends and family on health outcomes in older adults. Am. J. Health Promot..

[14] Rowe J.W., Fulmer T., Fried L. (2016). Preparing for better health and health care for an aging population. Jama.

[15] Shin J.K., Kim K.W., Park J.H., Lee J.J., Huh Y., Lee S.B., Choi E.A., Lee D.Y., Woo J.I. (2008). Impacts of poor social support on general health status in community-dwelling Korean elderly: The results from the Korean longitudinal study on health and aging. Psychiatry Investig..

[16] Ichida Y., Hirai H., Kondo K., Kawachi I., Takeda T., Endo H. (2013). Does social participation improve self-rated health in the older population? A quasi-experimental intervention study. Soc. Sci. Med..

[17] de Belvis A.G., Avolio M., Spagnolo A., Damiani G., Sicuro L., Cicchetti A., Ricciardi W., Rosano A. (2008). Factors associated with health-related quality of life: The role of social relationships among the elderly in an Italian region. Public Health.

[18] Abu Hammattah A., Mohd Yunus R., Matthias Müller A., Bahyah Kamaruzzaman S., Naqiah Hairi N. (2021). Association between structural social support and quality of life among urban older Malaysians. Australas. J. Ageing.

[19] Hamren K., Chungkham H.S., Hyde M. (2015). Religion, spirituality, social support and quality of life: Measurement and predictors CASP-12 (v2) amongst older Ethiopians living in Addis Ababa. Aging Ment. Health.

[20] Kooshiar H., Yahaya N., Hamid T.A., Abu Samah A., Sedaghat Jou V. (2012). Living arrangement and life satisfaction in older Malaysians: The mediating role of social support function. PLoS ONE.

[21] Mohamad Fuad M.A., Yacob H., Mohamed N., Wong N.I. (2020). Association of sociodemographic factors and self-perception of health status on oral health-related quality of life among the older persons in Malaysia. Geriatr. Gerontol. Int..

[22] Kim B.-R., Hwang H.-H. (2022). Analysis of Major Factors Affecting the Quality of Life of the Elderly in Korea in Preparation for a Super-Aged Society. Int. J. Environ. Res. Public Health.

[23] Altay B., Çavuşoğlu F., Çal A. (2016). The factors affecting the perception of elderly patients towards health, quality of life and health-related quality of life. TAF Prev. Med. Bull..

[24] Choi H.J., Lee H.J. (2019). A study on factors affecting quality of life for the elderly: Focusing on socio-demographic, environmental, and institutional characteristics. J. Korean Soc. Wellness.

[25] Jung J.P., Lee E.R., Sin M.S. (2012). The health of the elderly impact on quality of life. J. Korean Acad. Health Welf. Elder..

[26] Kim J.Y., Lee S.G., Lee S.K. (2010). The relationship between health behaviors, health status, activities of daily living and health-related quality of life in the elderly. J. Korean Gerontol. Soc..

[27] Bhatia R., Hirsch C., Arnold A.M., Newman A.B., Mukamal K.J. (2023). Social networks, social support, and life expectancy in older adults: The Cardiovascular Health Study. Arch. Gerontol. Geriatr..

[28] Schafer M.H., Sun H., Lee J.A. (2022). Compensatory connections? Living alone, loneliness, and the buffering role of social connection among older American and European adults. J. Gerontol. Ser. B.

[29] Mancini J.A., Quinn W., Gavigan M.A., Franklin H. (1980). Social network interaction among older adults: Implications for life satisfaction. Human Relat..

[30] Raggi A., Corso B., Minicuci N., Quintas R., Sattin D., De Torres L., Chatterji S., Frisoni G.B., Haro J.M., Koskinen S. (2016). Determinants of quality of life in ageing populations: Results from a cross-sectional study in Finland, Poland and Spain. PLoS ONE.

[31] Condello G., Capranica L., Migliaccio S., Forte R., Di Baldassarre A., Pesce C. (2019). Energy balance and active lifestyle: Potential mediators of health and quality of life perception in aging. Nutrients.

[32] Kalınkara V., Ceylan H. (2016). Population Aging and Its Social Dimensions, Sociology of Aging.

[33] Karasar N. (2013). Scientific Research Methodology.

[34] Diamond J.J., Becker J.A., Arenson C.A., Chambers C.V., Rosenthal M.P. (2007). Development of a scale to measure adults’ perceptions of health: Preliminary findings. J. Community Psychol..

[35] Lubben J.E. (1988). Assessing social networks among elderly populations. Fam. Community Health.

[36] Power M., Quinn K., Schmidt S., Group W.-O. (2005). Development of the WHOQOL-old module. Qual. Life Res..

[37] Kadıoglu H., Yıldız A. (2012). Validity and Reliability of Turkish Version of Perception of Health Scale. Türkiye Klinikleri. Tip Bilim. Derg..

[38] Demir Erbil D., Hazer O.Y.A. (2020). Adaptation of “Lubben Social Network Scale-6 (LSSAS-6)” to Turkish Culture: Validity and Reliability Study. Soc. Ment. Res. Think. J..

[39] Eser S., Saatli G., Eser E., Baydur H., Fıdaner C. (2010). The Reliability and Validity of the Turkish Version of the World Health Organization Quality of Life Instrument-Older adults Module (WHOQOL-Old). Turk. J. Psychiatry.

[40] Bayram N. (2010). Introduction to Structural Equation Modeling AMOS Applications.

[41] Hayes A.F. (2017). Introduction to Mediation, Moderation, and Conditional Process Analysis: A Regression-Based Approach.

[42] Ward M., McGarrigle C.A., Kenny R.A. (2019). More than health: Quality of life trajectories among older adults—Findings from The Irish Longitudinal Study of Ageing (TILDA). Qual. Life Res..

[43] Keidser G., Seeto M. (2017). The influence of social interaction and physical health on the association between hearing and depression with age and gender. Trends Hear..

[44] Santini Z.I., Koyanagi A., Tyrovolas S., Haro J.M., Fiori K.L., Uwakwa R., Thiyagarajan J.A., Webber M., Prince M., Prina A.M. (2015). Social network typologies and mortality risk among older people in China, India, and Latin America: A 10/66 Dementia Research Group population-based cohort study. Soc. Sci. Med..

[45] Park N.S., Jang Y., Chiriboga D.A., Chung S. (2019). Social network types, health, and well-being of older Asian Americans. Aging Ment. Health.

[46] Condello G., Capranica L., Stager J., Forte R., Falbo S., Di Baldassarre A., Segura-Garcia C., Pesce C. (2016). Physical activity and health perception in aging: Do body mass and satisfaction matter? A three-path mediated link. PLoS ONE.

[47] Ciaccioni S., Pesce C., Forte R., Presta V., Di Baldassarre A., Capranica L., Condello G. (2022). The Interlink among age, functional fitness, and perception of Health and quality of life: A mediation analysis. Int. J. Environ. Res. Public Health.

